# Prey-switching does not protect a generalist turtle from bioenergetic consequences when its preferred food is scarce

**DOI:** 10.1186/s12898-020-00279-6

**Published:** 2020-02-18

**Authors:** Kristen Petrov, Ricky-John Spencer, Natasha Malkiewicz, Jessica Lewis, Claudia Keitel, James U. Van Dyke

**Affiliations:** 1 School of Science, Hawkesbury Institute, Western Sydney University, Locked Bag, 1797, Penrith, NSW 2751 Australia; 2 School of Life and Environmental Sciences, University of Sydney, 380 Werombi Road, Brownlow Hill, NSW 2570 Australia; 3 School of Molecular Sciences, La Trobe University, Albury-Wodonga Campus, PO Box 821, Wodonga, VIC 3689 Australia

**Keywords:** Optimal foraging, Body condition, Food webs, Environmental change, Stable isotopes

## Abstract

**Background:**

Optimal foraging theory explains how animals make foraging decisions based on the availability, nutritional content, and handling times of different food types. Generalists solve this problem by consuming a variety of food types, and alter their diets with relative ease. Specialists eat few food types, and may starve if those food types are not available. We integrated stable isotope analyses with previously-published stomach contents and environmental data to investigate how the foraging ecologies of three sympatric freshwater turtle species vary across four wetlands that differ in turbidity and primary producer abundance.

**Results:**

We found that the generalist *Emydura macquarii* consumes a varied diet (but mostly filamentous green algae) when primary producers are available and water is clear, but switches to a more carnivorous diet when the water is turbid and primary producers are scarce, following the predictions of optimal foraging theory. In contrast, two more-specialized carnivorous species, *Chelodina expansa* and *Chelodina longicollis*, do not differ in diet across wetlands, and interspecific competition may increase where *E. macquarii* is carnivorous. When forced to be more carnivorous, *E. macquarii* exhibits higher rates of empty stomachs, and female turtles have reduced body condition, but neither *Chelodina* species are affected.

**Conclusions:**

Our results provide support for optimal foraging theory, but also show that the ability to change diet does not protect the generalist from experiencing lower foraging success when its preferred food is rare, with direct consequences for their energy budgets. Our results have conservation implications because wetlands in the Murray–Darling river system are increasingly turbid and have low macrophyte abundance, and all three species are declining.

## Background

Optimal foraging theory explains how animals make foraging decisions based on the nutritional content of prey, prey abundance, and intra- or interspecific competition [33, 34, 41]. An animal is predicted to select prey based on the fitness benefits the animal will receive by consuming that prey compared to others [27]. In the simplest circumstances, animals select prey that will maximize the rate of energy and nutrient intake against the cost of searching for and/or handling food [33]. Environmental change can alter the availability of preferred or optimal prey and cause a shift in prey selection. For example, surgeonfish (*Acanthurus nigrofuscus*) primarily consume brown and red algae in summer, but switch their diet in winter to green algae as it becomes more abundant [16]. Masked palm civets (*Paguma larvata*) alter their diet across temporal and spatial scales, depending on the availability of fruit. They eat fruit when it is readily available, but eat small mammals when it is not [49]. How animals change their foraging in response to changes in food abundance is likely to incur costs that are under natural selection [15] and vary among generalist and specialist species. For generalist species that consume a variety of prey, the loss of a “most-preferred” prey species due to environmental change is unlikely to impact their foraging efficiency because they can theoretically expand their diets to include something else [42]. For species that specialize on only one (or a few) prey species, the loss of a preferred prey item can reduce foraging efficiency [19], and thus reduce their energy intake.

Turtles are good model taxa for studying the effects of temporal changes in diet. Most species consume a diversity of prey and have long lifespans, so the temporal effects of changes in diet can be studied within individuals. Three species of freshwater turtles inhabit the Murray River, Australia, and vary between diet specialization and generalization: the broad-shelled turtle (*Chelodina expansa*), common long-necked turtle (*Chelodina longicollis*), and Murray River short-necked turtle (*Emydura macquarii*). *Chelodina expansa* and *C. longicollis* are specialist and generalist carnivores, respectively. *Chelodina expansa* preys on crustaceans, fish, and carrion, whilst *C. longicollis* preys on invertebrates, small fish, and crustaceans [6, 7]. *Emydura macquarii* is a generalist omnivore, which consumes algae, carrion, and invertebrates [8, 39, 40].

We have previously compared the stomach contents of *C. expansa, C. longicollis,* and *E. macquarii* at four sites in north-central Victoria [29]. These wetlands differ broadly in turbidity, and in plant and invertebrate communities [29]. *Chelodina expansa*, *C. longicollis*, and *E. macquarii* are sympatric in these wetlands, and stomach contents data indicate that they broadly overlap in the food they consume [29]. The stomach contents of both *Chelodina* species do not differ across sites, whereas *E. macquarii* stomach contents vary considerably with location, and largely reflect the availability of prey within a given wetland, which differed in association with turbidity and macrophyte diversity [29]. These results were consistent with optimal foraging theory since the generalist (*E. macquarii*) should be expected to be more capable of changing its diet in response to changes in food abundance than the more specialized *Chelodina* species. However, stomach contents data offer only snapshots of information about animal diets, and these data alone could not demonstrate whether the differences we observed are temporally consistent, or whether they have consequences for the life histories of these species.

Here, we used stable isotope analysis to determine whether turtle diets vary consistently over time across site and among species, based on the environmental and stomach-contents differences identified previously in Petrov et al. [29]. We then compared turtle body conditions to test whether long-term trends in diet impacted on turtle life histories. Stable isotope analyses are powerful tools for testing for spatial and temporal variation in diet, and interspecific competition [17, 26, 48]. We used δ^13^C to trace sources of primary production underlying the food webs each turtle species relied on [22, 23]. We used δ^15^N to compare the relative trophic position each turtle species occupied within each wetland, assuming that δ^15^N of each turtle should be enriched by 3–4‰ relative to its diet, following Post [32]. Specifically, we tested: (1) whether the three species consistently overlap in diet, which could result in competition for food; (2) whether stable isotope and stomach contents data provide consistent inferences about turtle diets; (3) whether any species exhibits diet differences across different wetlands; and (4) whether apparent diet differences across site have consequences for body condition of any species.

## Results

Between February–March 2015 we caught a total of 20 *C. expansa*; 12 *C. longicollis* and 90 *E. macquarii*. The following season between December 2015–March 2016 we caught a total of 13 *C. expansa*; 13 *C. longicollis*; and 83 *E. macquarii* (Table 1). There were no recaptures in this study.

**Table 1:** Numbers of each turtle species caught per sampling period, across wetlands

	Cockatoo	Gunbower Creek	Longmore	Safes
February–March 2015				
*Chelodina expansa*	11	2	5	2
*Chelodina longicollis*	9	1	2	0
*Emydura macquarii*	30	12	14	34
December 2015–March 2016				
*Chelodina expansa*	1	4	4	4
*Chelodina longicollis*	3	3	1	7
*Emydura macquarii*	16	21	30	16

### Primary producer isotopic compositions

**Fig. 1:**
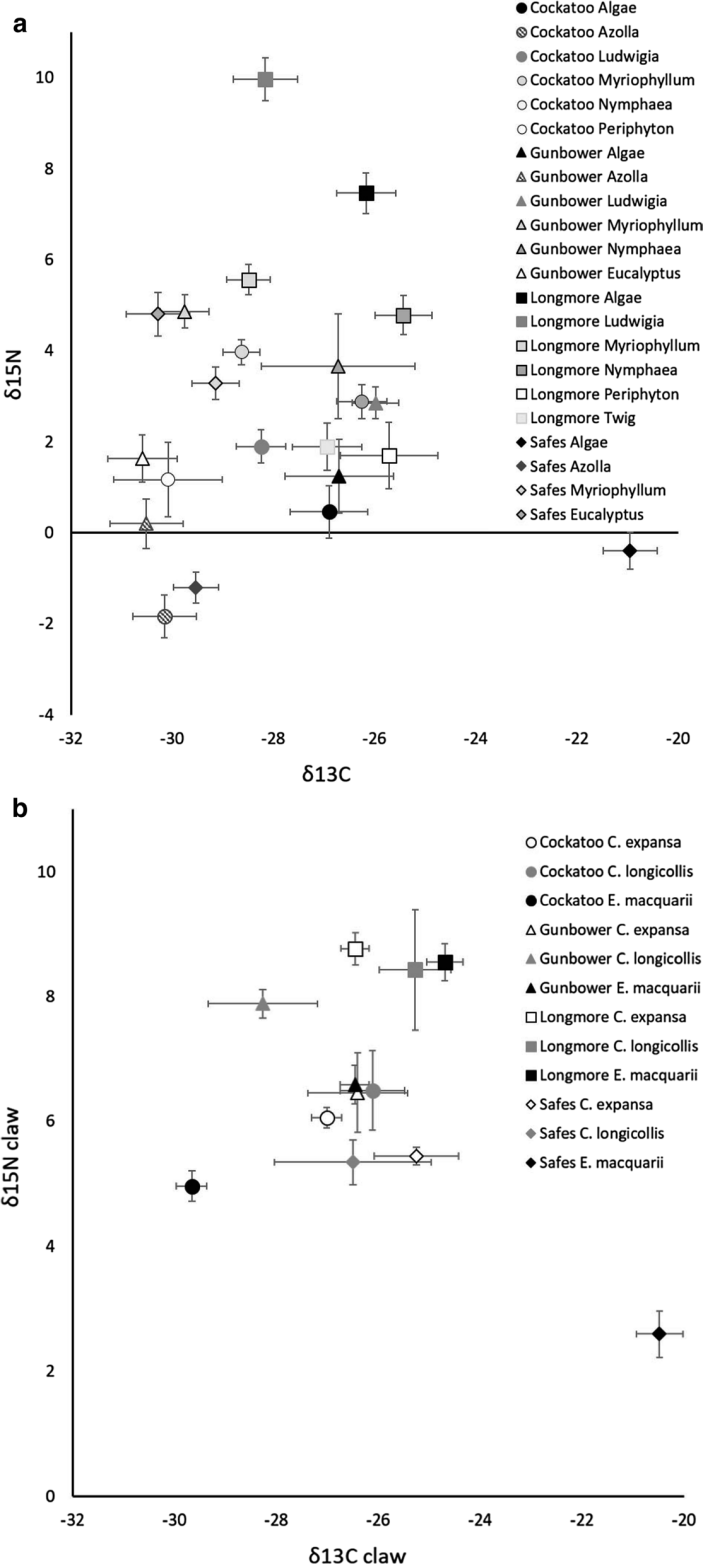
Isotopic compositions of **a** primary producers and **b** turtle claws from each wetland

Primary producer isotopic compositions (δ^13^C and δ^15^N) differed across an interaction of site × species (Table 2, *P* < 0.001). Univariate analyses of δ^13^C and δ^15^N showed that the interaction effect was driven by both δ^13^C (*F*_*11, 298*_ = 9.26, *P* < 0.001) and δ^15^N (*F*_*11, 298*_ = 25.32, *P* < 0.001). Thus, most primary producer species varied uniquely in both δ^13^C and δ^15^N across each wetland (Fig. 1), and wetland differences at the base of the food web need to be standardized before turtle isotopic composition can be compared across wetlands.

**Table 2:** Results of MANOVA analysis of mean plant δ^13^C and δ^15^N sampled from the four wetlands

Effect	Pillai’s trace	*F*	Num *df*	Den *df*	*P*
Site*	0.620	44.59	6	596	< 0.001
Species*	0.949	38.41	14	596	< 0.001
Site × species*	0.696	14.45	22	596	< 0.001

Asterisks indicate significant effects

### Primary producer vs turtle δ^13^C

Mean δ^13^C of primary producers and turtles differed uniquely across sites (*F*_*33, 159*_ = 51.57, *P* < 0.001; Fig. 1). At Cockatoo Lagoon, δ^13^C of *C. expansa* and *E. macquarii* were similar to the δ^13^C of all of the primary producers present (all *P* > 0.184, Table 3), while δ^13^C of *C. longicollis* was similar only to the δ^13^C of filamentous green algae (*P* = 0.999), *Ludwigia* (*P* = 0.989), *Myriophyllum* (*P* = 0.944), *Nymphaea* (*P* = 0.999) and periphyton (*P* = 0.778), and was dissimilar to *Azolla* (*P* = 0.033, Table 3). At Gunbower Creek, δ^13^C of *C. expansa*, *C. longicollis* and *E. macquarii* was similar to δ^13^C of the primary producers present (all *P* > 0.097, Table 3). At Longmore Lagoon, δ^13^C of both *Chelodina* species was similar to δ^13^C of the primary producers (all *P* > 0.940), while δ^13^C of *E. macquarii* was only similar to filamentous green algae (*P* = 0.581), *Nymphaea* (*P* = 0.999), periphyton (*P* = 0.984) and twig (*P* = 0.320) and dissimilar to *Ludwigia* (*P *= 0.003), and *Myriophyllum* (*P *= 0.002, Table 3). At Safes Lagoon, δ^13^C of both *Chelodina* species was similar to δ^13^C of the primary producers (all *P* > 0.093), δ^13^C of *E. macquarii* was similar only to filamentous green algae (*P* = 0.999) and dissimilar to *Azolla* (*P *< 0.001), *Myriophyllum* (*P *< 0.001) and *E. camaldulensis* (*P *< 0.001, Table 3).

**Table 3:** Plants that did not differ from turtle δ^13^C were considered food web bases for each species/wetland

	Cockatoo	Gunbower	Longmore	Safes
*C. expansa*	Algae	Algae	Algae	Algae
	*Azolla*	*Azolla*	*Ludwigia*	*Azolla*
	*Ludwigia*	*Ludwigia*	*Myriophyllum*	*Myriophyllum*
	*Myriophyllum*	*Myriophyllum*	*Nymphaea*	*Eucalyptus*
	*Nymphaea*	*Nymphaea*	Periphyton	
	Periphyton	*Eucalyptus*	Twig	
*C. longicollis*	Algae	Algae	Algae	Algae
	*Ludwigia*	*Azolla*	*Ludwigia*	*Azolla*
	*Myriophyllum*	*Ludwigia*	*Myriophyllum*	*Myriophyllum*
	*Nymphaea*	*Myriophyllum*	*Nymphaea*	*Eucalyptus*
	Periphyton	*Nymphaea*	Periphyton	
		*Eucalyptus*	Twig	
*E. macquarii*	Algae	Algae	Algae	Algae
	*Azolla*	*Azolla*	*Nymphaea*	
	*Ludwigia*	*Ludwigia*	Periphyton	
	*Myriophyllum*	*Myriophyllum*	Twig	
	*Nymphaea*	*Nymphaea*		
	Periphyton	*Eucalyptus*		

### Turtle isotopic compositions

After correcting for food web base differences, turtle estimated trophic position (ETP) did not differ with straight carapace length (SCL), sex, site, species, or across the full factorial interactions (Additional file 1: Table S1). We ran a reduced model without the interactions of the fixed effects and SCL, that only tested for differences in the intercepts of the relationships between isotope and SCL, rather than differences in slope. We removed sex from the model because we did not have large enough sample sizes of each sex for each species of turtle within each wetland to perform post hoc tests on significant factors when sex was included. In the reduced MANCOVA, turtle ETP differed significantly among species (Table 4, *F*_*4, 280*_ = 17.24, *P *< 0.001*)* and site (Table 4, *F*_*6, 280*_ = 9.37, *P *< 0.001). Univariate analyses of claw and skin showed that the multivariate differences occurred in both claw (site: *F*_*3, 140*_ = 13.37, *P* < 0.001; species *F*_*2, 140*_ = 5.79, *P* = 0.004) and skin (site; *F*_*3, 140*_ = 20.07, *P* < 0.001; species *F*_*2, 140*_ = 43.03, *P* < 0.001).

**Table 4:** Results of MANOVA analysis of differences in ETP from claw and skin samples

Effect	Pillai’s trace	*F*	Num *df*	Den *df*	*P*
SCL	0.020	1.45	2	139	0.238
Site*	0.335	9.37	6	280	< 0.001
Species*	0.395	17.24	4	280	< 0.001
Site × species	0.100	1.23	12	280	0.261

Asterisks indicate significant effects

**Fig. 2:**
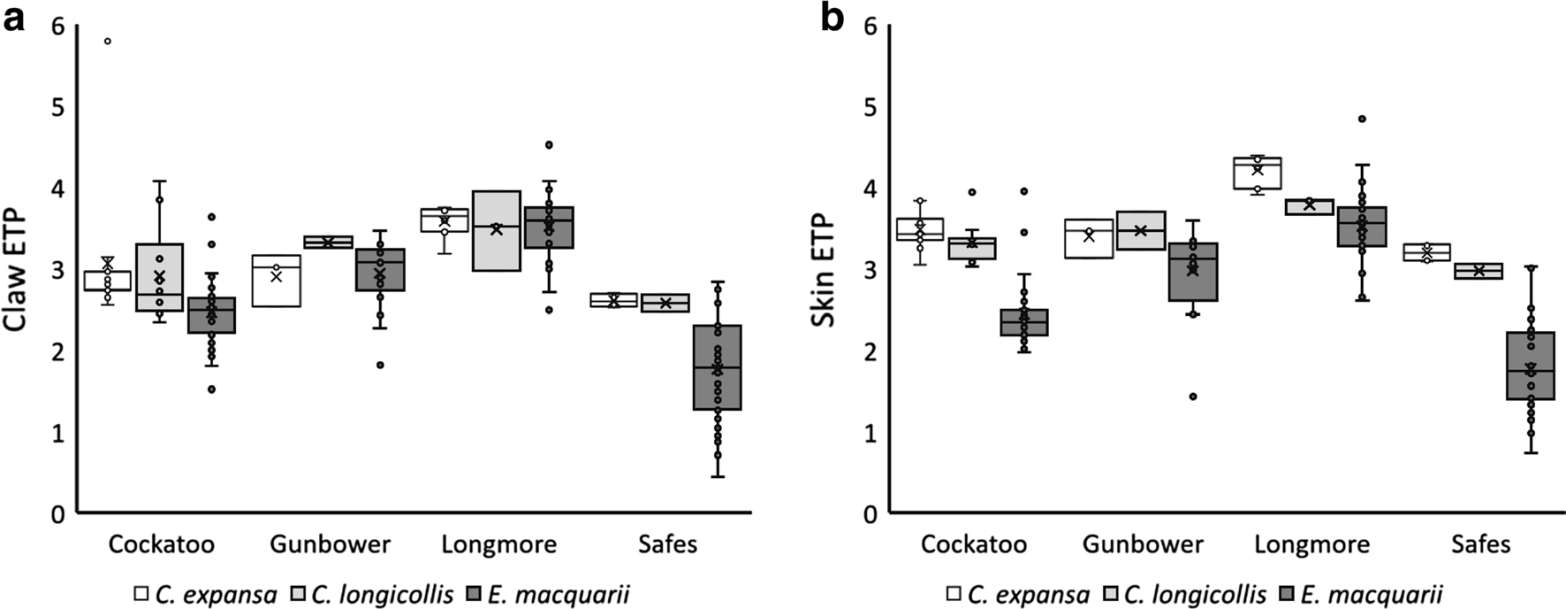
ETP from **a** claw and **b** skin for *C. expansa*, *C. longicollis*, and *E. macquarii*

The among-species differences occurred because the ETP of *E. macquarii* were lower than the ETP of *C. expansa* (claw *P *= 0.035; skin *P* < 0.001) and were marginally lower than *C. longicollis* (claw *P *= 0.049; skin *P* < 0.001). The difference between *C. longicollis* and *E. macquarii* was more pronounced in skin samples than in claw samples (Fig. 2). In contrast, *C. expansa* and *C. longicollis* ETP did not differ in either skin or claw samples (claw *P *= 0.246; skin *P* = 0.910; Fig. 2). The lack of a significant site-by-species interaction here is possibly due to low sample sizes for the *Chelodina* species, particularly at Gunbower Creek and Longmore Lagoon. With a larger sample size, it is possible that we would have observed a significant interaction in the claw data because there is no apparent difference in the claw ETP across species within these two sites (Fig. 2a). Likewise, some *E. macquarii* had relatively high ETP at each site, which was comparable to those of the *Chelodina* species, but most *E. macquarii* ETP tended to be lower than those of the other species (Fig. 2a).

The among-site differences in ETP were consistent regardless of tissue type and species (Fig. 2). Turtles from Safes Lagoon had ETP that were significantly lower than turtles from the other three sites (claw and skin *P *< 0.007). Turtles from Longmore Lagoon had significantly higher ETP than turtles from the other three sites (claw and skin *P* < 0.005). Turtles from Cockatoo Lagoon and Gunbower Creek had intermediate ETP that did not differ from each other (skin and claw *P* > 0.785). These differences were largely driven by *E. macquarii*, since the capture rates for both *Chelodina* species were low within each wetland.

### ETP consistency between claw and skin

**Fig. 3:**
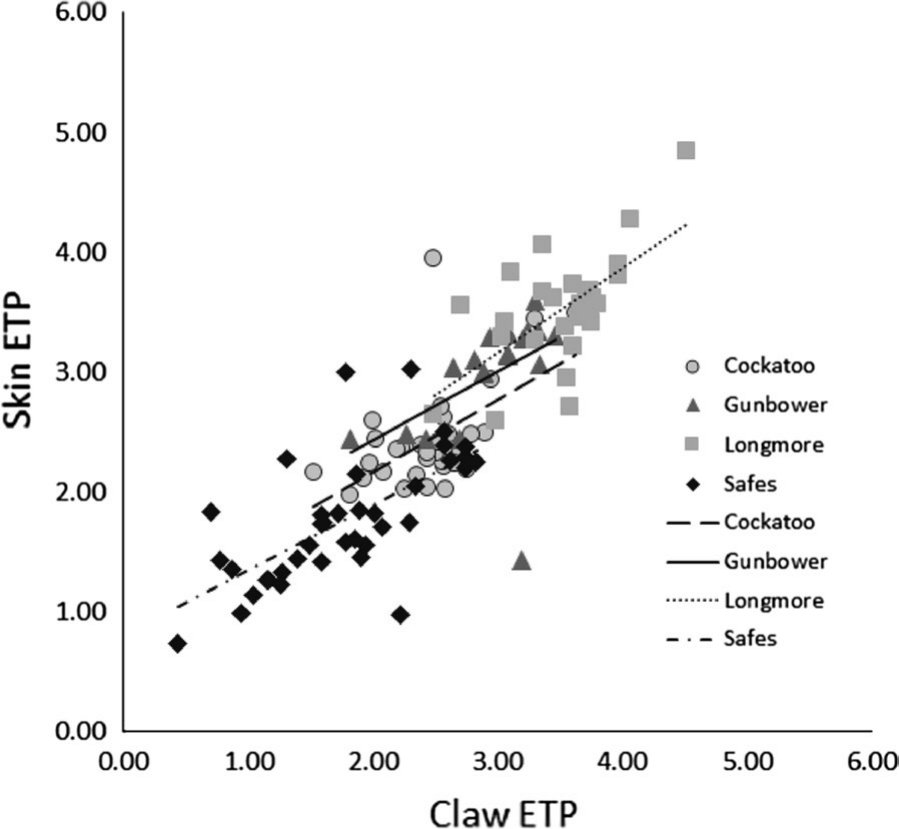
Relationships between the ETP of claw and skin samples in *E. macquarii*, from each site

*Emydura macquarii* skin ETP increased linearly with claw ETP (*F*_*1, 106*_ = 57.4, *P* < 0.001) and differed across sites (*F*_*3, 106*_ = 7.48, *P* = 0.001; Fig. 3). There was no interaction between claw ETP and site (*F*_*3, 103*_ = 0.35, *P* = 0.787), so the slopes of the relationships were the same regardless of site (Fig. 3, R^2^ for each site were: Cockatoo = 0.32, Gunbower Creek = 0.22, Longmore = 0.40, Safes = 0.43). After accounting for the effect of claw ETP, the skin ETP of turtles from Longmore Lagoon was higher than that of Cockatoo Lagoon and Safes Lagoon (*P *< 0.004; Fig. 3), but not Gunbower Creek (*P *= 0.281). The skin ETP of turtles from Safes Lagoon was also lower than that of turtles from Cockatoo Lagoon and Gunbower Creek (*P *< 0.025; Fig. 3). Together, these results indicate that *E. macquarii* from Longmore Lagoon have consistently high ETP, whereas *E. macquarii* from Safes Lagoon have consistently low ETP, and *E. macquarii* from Gunbower Creek and Cockatoo Lagoon have consistently intermediate ETP (Fig. 3). We did not compare claw and skin ETP in either *Chelodina* species due to the low number of turtles we caught within each site.

### Turtle stomach contents and isotopic estimated trophic position

**Fig. 4:**
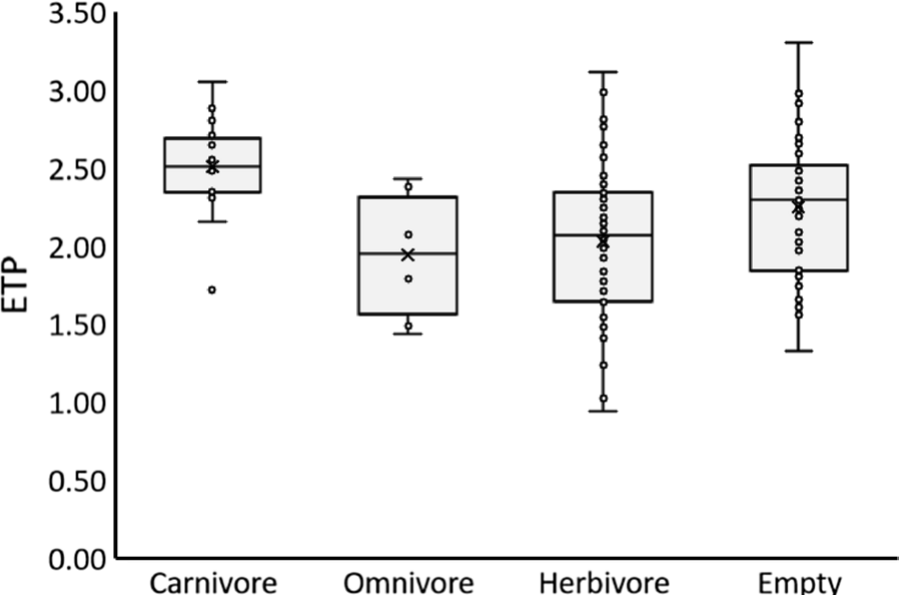
ETP was higher for carnivorous turtles than for omnivorous and herbivorous turtles

Estimated trophic position differed among turtles classed with different stomach contents (*F*_*3, 108*_ = 5.78, *P* = 0.001), across all sites and species. This difference was driven by carnivorous turtles, which had higher mean ETP than did herbivorous or omnivorous turtles (Fig. 4; *P* < 0.019). Turtles with empty stomachs did not differ in ETP from any other group and exhibited an intermediate ETP. Herbivorous and omnivorous turtles did not differ from each other (Fig. 4; *P* > 0.119).

### Body condition

**Fig. 5:**
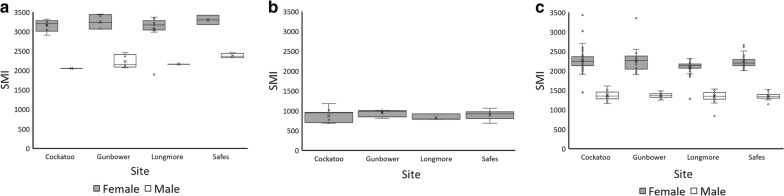
SMI for **a***Chelodina expansa*, **b***Chelodina longicollis*, and **c***Emydura macquarii*, across sites

Body conditions did not vary across wetland in male (*F*_*3, 11*_ = 3.26, *P* = 0.081) or female *C. expansa* (Fig. 5a *F*_*3, 20*_ = 0.54, *P* = 0.661), or in *C. longicollis* where sexes were combined (Fig. 5b; *F*_*3, 24*_ = 0.44, *P* = 0.730). Body conditions of female *E. macquarii*, were significantly different across the four wetlands of our study (*F*_*3, 165*_ = 5.22, *P* = 0.002). Post-hoc tests revealed that female *E. macquarii* from Longmore Lagoon had lower SMI than female turtles from the other three sites (Fig. 5c; *P* < 0.029). In contrast, male *E. macquarii* body condition did not differ across wetlands (*F*_*3, 118*_ = 0.34, *P *= 0.793).

## Discussion

Our analysis provides robust evidence of how foraging ecology of three sympatric freshwater turtle species is impacted by environmental differences, with consequences for both interspecific competition and individual energy budgets. Taken alongside our previous environmental comparisons [29], our data indicate that turbidity and diversity of primary producers (macrophytes, filamentous green algae) impact both short- and long-term foraging by turtles, particularly the generalist *E. macquarii*. Where aquatic primary producers are scarce and turbidity is high (Longmore Lagoon), *E. macquarii* had a more carnivorous diet, based on stomach contents [29] and stable isotope data (this study). In contrast, where aquatic primary producers are common and water is clear (Safes Lagoon), *E. macquarii* had a more herbivorous diet, and at two intermediately turbid locations (Cockatoo Lagoon and Gunbower Creek), *E. macquarii* exhibited an omnivorous diet [29]. High turbidity can limit primary producer photosynthesis [25] and alter primary producer diversity [37], a trend which was apparent in our previous study at Longmore Lagoon. Our previous study also showed that Longmore Lagoon *E. macquarii* had empty stomachs more frequently, and many turtles contained wood in their stomachs [29], which is not digestible. Turtles consuming wood would likely have a higher δ^15^N than would be expected from the wood itself, as they would be only able to digest and absorb the bacteria and fungi that were decomposing the wood. Thus, they would likely resemble second-order consumers isotopically [32], which could contribute to their relatively carnivorous ETP. Likewise, the relatively high ETP of *E. macquarii* at Longmore could be a result of starvation, which can cause an increase in δ^15^N of consumers [12, 13], because they often had empty stomachs [29].

In contrast to *E. macquarii*, stable isotope data indicated that both *C. expansa* and *C. longicollis* exhibited consistently carnivorous diets, regardless of location. These results align with our stomach contents observations, which identified predominately animal prey for both *C. expansa* and *C. longicollis* [29]. Thus, the diets of all three species in our study largely reflect results of past stomach contents analyses [6–8].

Our analysis adds to past studies in a robust way because we show consistent temporal trends in diet data, particularly for *E. macquarii* [8, 29]. Stomach contents data represent what a turtle has consumed in the past several hours, whereas skin stable isotope data represent what a turtle has consumed in the past 3–6 months [36], and claw stable isotope data represent what a turtle has consumed over the past ~ 12 months [1, 2, 38]. Despite these temporal differences, among-site differences in ETP from skin were consistently reflected in claw ETP in *E. macquarii*. Thus, stable isotope results alone indicate that *E. macquarii* ETP is consistent over both medium and long timespans, within each wetland. Likewise, our comparison of stomach contents and stable isotope data, though coarse, suggests that turtles (of all three species) with carnivorous stomach contents have consistently higher ETPs, whereas turtles with herbivorous or omnivorous stomach contents have lower ETPs. Turtles with empty stomachs had intermediate ETP, but most of these would have come from Longmore Lagoon and Gunbower Creek. Thus, their combined ETP would have been lower than expected from Longmore Lagoon alone, and vice versa for turtles from Gunbower Creek.

The overlaps in ETP suggest that there is potential for interspecific competition between *Chelodina* species, and both might compete with *E. macquarii* for animal prey. However, the within-site variation in δ^13^C we observed in all three species shows that even though all three species can be carnivorous, they consume a variety of animal prey that differs across sites. The δ^13^C variation reflects stomach contents data, which although limited in sample size for the *Chelodina* species, identified 14 different prey items in *C. longicollis* (mostly small arthropods) and 9 in *C. expansa* (crayfish, fish, and small arthropods; [29]). *Emydura macquarii* did not contain any animal species not found in *Chelodina* [29]. The diversity in invertebrate prey *within* wetland is likely to obscure our stable isotope analysis [29]. The invertebrate prey items we identified often overlapped in isotopic composition, such that the isotopic composition of a turtle could reflect multiple possible prey that we cannot distinguish [30]. Thus our evidence for interspecific competition may be equivocal. We suggest the *potential* for competition is highest in Longmore Lagoon, where primary producers are scarce and *E. macquarii* consumes more animal prey, and lowest in Safes and Cockatoo Lagoons, where primary producers are more abundant and *E. macquarii* is more herbivorous [29]. The risk of interspecific competition may drive across-wetland movement often observed in *C. longicollis* [20]. We assume that the turtles we captured, except *C. longicollis*, do not travel between sites, because of the distances between them (≥ 9.15 km by river; [29]).

Our results show how generalist species can change their diets when preferred prey are scarce, following the predictions of optimal foraging theory [33]. However, our results also show that the ability to switch diets does not protect a generalist species from bioenergetic consequences of the absence of its preferred food. Where filamentous green algae are abundant, *E. macquarii* maintains a varied yet largely herbivorous diet that likely limits competition with the carnivorous *Chelodina* spp. Where algae are scarce, *E. macquarii* adds alternative animal prey to their diet, which may increase the risk of competition with the carnivorous *Chelodina* spp. Female *E. macquarii* exhibit reduced body condition under these conditions, whereas neither of the more specialized *Chelodina* spp. do. Estimates of body condition do not necessarily represent fat mass alone in turtles [11], so we assume that the differences in SMI we detected represent a composite of reduced fat, muscle, and/or visceral mass.

There are several possible explanations for why body condition differences only occurred in female *E. macquarii.* First, *E. macquarii* has difficulty catching mobile prey [39], and may have a competitive disadvantage to both *Chelodina* spp., which forage on mobile animals [6, 7]. The large number of empty stomachs in *E. macquarii* from Longmore Lagoon, where algae is scarce [29], supports this interpretation. Reduced foraging success lowers the amount of energy *E. macquarii* can allocate to various biological demands (e.g., [4, 14]). Second, adult *E. macquarii* have the highest resting metabolic rates of the three species [10], and are most susceptible to losing body mass due to low foraging success. The lack of a difference in male body condition may be because males do not invest in egg production [44]. It is possible that we did not detect body condition differences in either *Chelodina* species because of our lower catch rates of both species, which may also reflect small population densities within our study system, compared to *E. macquarii*.

## Conclusions

Our results have implications for turtle and freshwater conservation. *Emydura macquarii* and *C. longicollis* are in decline across their range, while *C. expansa* is listed as endangered [9, 46, 47]. Much of the Murray–Darling river system is degraded [35], and many wetlands have been lost due to flow regulation by dams [21], or drought [24]. Approximately 80% of the wetlands that remain have been described as highly turbid and depauperate in primary producer abundance [18], similar to Longmore Lagoon [29]. Thus, environmental differences in turtle foraging ecology, and their bioenergetic consequences, are likely to be widespread in the Murray–Darling system. We suggest that other species within the food web of the Murray–Darling may be similarly affected, and further studies are needed to determine the biotic consequences of environmental degradation. Likewise, our results broadly show how environmental factors, like turbidity and food abundance, can drive changes in foraging ecology of freshwater animals that vary between specialism and generalism. Notably, our results show that even generalist species, which can theoretically forage on alternatives as prey abundances change [42], cannot completely escape the bioenergetic effects of a scarce preferred prey species. The loss of preferred prey species force animals to make foraging decisions that will ultimately dictate their energy intake and life histories [3, 14]. Finally, our results further validate the accuracy of stable isotopes (against stomach contents) for studying foraging ecology in animals.

## Methods

### Study sites

We studied turtle diets at four sites adjacent to the Murray River between Cohuna and Gunbower, Victoria, Australia: Cockatoo Lagoon, Gunbower Creek, Longmore Lagoon and Safes Lagoon, in February–March 2015 and December 2015–March 2016. Cockatoo, Longmore, and Safes Lagoons are wide-bodied oxbow lakes that are connected to Gunbower Creek, and all four sites vary widely in turbidity and primary producer community [29]. These sites were selected based on a previous study that compared stomach contents of each turtle species across the four wetlands [29]. Full site descriptions can be found in Petrov et al. [29], but Longmore Lagoon had the highest turbidity and lowest primary producer abundance, whilst Safes Lagoon had the lowest turbidity and highest primary producer abundance [29]. Cockatoo Lagoon and Gunbower Creek were both intermediate [29].

### Isotope sampling

In 2015 and 2016, we opportunistically collected at least 10 samples from all macrophyte species present in each wetland [29], as well as periphyton, filamentous green algae, and terrestrial river red gum (*Eucalyptus camaldulensis*), which often overhangs the bank. We spread our sampling randomly across each wetland by walking or canoeing at least 5 m, in a randomly-chosen direction, between each sample. If a species was rare, we collected a sample each time we saw an individual. All samples were frozen and stored at − 20 °C prior to analysis.

In 2015 and 2016, we trapped all turtle species using baited cathedral traps, fyke nets, and collapsible crab traps. We carried out trapping to obtain study turtles for the purpose of sampling for stable isotope analyses, rather than comparing species population densities. We set traps at least 5 m apart and checked them every 10–14 h. In both 2015 and 2016, we trapped each site with 4–8 traps until a minimum of 15 and maximum of 30 turtles of the most-common species were caught. Traps were set continuously for at least 5 days, up to a maximum of 20 trap-days, depending on capture rates. In total, the sites were trapped for an average of 1500 trap-hours.

Turtles caught were identified, sexed, weighed (to the nearest g) and measured (carapace and plastron length and width; to the nearest mm; [40]). Males were distinguished from females by the lengths of their tails in *C. expansa* and *E. macquarii.* We tentatively determined the sex of *C. longicollis* by the concavity of the plastron in males [5]. Turtles were individually marked by notching marginal scutes. Juveniles were recorded as any turtle having a straight carapace length below 18 cm in *C. longicollis*, 22 cm in *C. expansa* and 19 cm in *E. macquarii* [9]. In both years, we collected claw clippings from each of the toes of the left hind leg and skin from the webbing between the 4th and 5th toes of the left hind leg of each turtle. We collected both claws and skin to compare two different relative timelines of foraging in each turtle: claws reflect the isotopic composition of the prey eaten over the past ~ 12 months, while skin reflects the isotopic composition of prey consumed over 3–6 months [1, 2, 36, 38]. All samples were frozen and stored at − 20 °C prior to analysis.

From December, 2015 to March, 2016, we also stomach-flushed all non-juvenile turtles to have instantaneous snapshots of foraging ecology (for details, see [29]). During this period, we only analyzed claw *or* skin isotope composition for the majority of turtles captured, because our 2015 isotope sample size was too large to analyze every sample. We chose to analyze skin or claw at random for each turtle, and randomly chose a subset of individuals (at least one quarter of the turtles caught at a site) to analyze both claw and skin isotopic compositions to control for between-year effects in environmental isotope fractionation.

### Stable isotope analyses

All samples were washed in deionized water and freeze-dried at − 40 °C to asymptotic mass using an Edwards Modulyo Freeze Dryer (Burgess Hill, United Kingdom). We homogenized the samples to powder in a Qiagen TissueLyser II (Hilden, Germany) and stored the samples in a desiccator until isotopic analysis. One mg of skin and claw homogenate, 1.65 mg of macrophytes and 3 mg of periphyton, twig, filamentous green algae, and redgum were weighed into tin capsules. Packaged samples were placed in 96-well microplates prior to analysis. Using a Thermo Scientific Delta V Advantage isotope ratio mass spectrometer (Waltham, Massachusetts, United States) coupled to a ConfloIV and FlashHT at the Centre for Carbon, Water and Food of the University of Sydney, δ^13^C and δ^15^N of the samples was determined. Samples sealed in tin capsules were loaded into an autosampler, which individually dropped them into a helium-flushed oxidation reactor at 1000 °C with an oxygen injection at sample drop resulting in the combustion of the samples. After passage through the oxidation reactor, the combustion gases (at this stage CO_2_, NO_x_ and H_2_O) were carried by helium through a reduction reactor converting NO_x_ to nitrogen gas, and subsequently passed through a drying agent to remove H_2_O. Nitrogen and carbon dioxide gases were separated by a gas chromatograph and transported into the isotope ratio mass spectrometer, which measured the mass to charge ratio of the different isotopologues (^12^C^16^O^16^O and ^13^C^16^O^16^O, ^14^N^14^N and ^14^N^15^N) of the sample combustion gases. Isotopic values are expressed in delta notation (‰), relative to VPDB for carbon and AIR for nitrogen. Precision was between 0.05 and 0.1‰ for both carbon and nitrogen analyses in each run (1 SD; n = 8).

### Statistical analyses

First, we tested whether the bases of the food webs differed in isotopic composition at each wetland, in order to test for wetland-specific isotopic differences. We compared primary producer (macrophyte, algae, redgum, and periphyton) mean isotopic compositions (δ^13^C and δ^15^N) using a multivariate analysis of variance (MANOVA) in SAS (PROC GLM; SAS Institute, Cary, NC), with site, species, and their interaction included as main effects, and year included as a random effect. We did not account for primary producer abundance or productivity in this analysis, as the abundance of primary producer species may affect their contribution to the isotopic signatures we observe, with rare species potentially making large contributions to the food web.

Second, we compared the mean δ^13^C of each primary producer species to the mean δ^13^C of each turtle species within each wetland, to determine which primary producer species most likely formed the main base(s) of the food web for each turtle species, within each wetland. Although δ^13^C enriches slightly with trophic position, the fractionation rate is generally much smaller than that of nitrogen [43], so we assumed that δ^13^C overlap between a turtle species and a primary producer species would indicate that species was likely to be a base to the turtle’s food web. Furthermore, overlap in δ^13^C across multiple primary producer species within a site would mean that we could isotopically distinguish which species was that base, such that some sites could have multiple primary producer species as the base of the food web. Thus, the aim of our test was to determine which primary producer species were not significantly different in δ^13^C from each turtle species within each site, and could be a “base” for that turtle species’ food web. We used this match-based approach because preliminary data indicated broad overlap in isotopic compositions of prey items, which a mixing model would not be able to discriminate [31].

To perform this analysis, we ran an ANOVA in SAS (PROC MIXED), with species nested within site as the main effect and year as a random effect. We then compared least-squares means of the primary producer and turtle δ^13^C using a Dunnett’s post hoc test. In this analysis, we averaged claw and skin δ^13^C values for each turtle.

Third, we estimated the relative trophic position (ETP) of each turtle using the following equation, from Post [32]:

$${\text{Estimated trophic position }} =\uplambda + \, (\updelta^{ 1 5} {\text{N}}_{\text{secondary consumer}} - \,\updelta^{ 1 5} {\text{N}}_{\text{base}} )/\Delta_{\text{n}}$$

where λ is the trophic position of the base (primary producer: 1); δ^15^N_secondary consumer_ is the isotopic value of the turtle tissue; δ^15^N_base_ is the mean isotopic value of the primary producer at the food web base, and Δ_n_ is the enrichment in δ^15^N per trophic level, here set as 3.4% following Post [32]. We chose the species of primary producers to include in our mean calculation of δ^15^N_base_ independently for each turtle species within each site. We chose primary producer species that were not significantly different in δ^13^C from a turtle species within a site, as determined in analysis 2 above. We then compared the calculated ETP of each turtle species across all four wetlands, using both claw and skin estimates, in a multivariate analysis of covariance (MANCOVA) in SAS (PROC GLM). The main effects included straight carapace length (SCL), sex, site, species, and full factorial interactions. We included year as a random effect.

Fourth, we used an ANCOVA (PROC MIXED) to test whether ETPs from skin and claw estimates were correlated, such that a turtle with a high ETP from claw also had a high ETP from skin. We used this approach to test the hypothesis that dietary differences across site were consistent over time, based on the differences in tissue turnover time between skin (3–6 months) and claw (~ 12 months; [1, 2, 38]). In the ANCOVA, skin ETP was the dependent variable, claw ETP was the covariate, and site was included as a main effect. We also included year as a random effect. If a turtle only had data for one tissue type, it was excluded from this analysis.

Fifth, we validated our ETP against the stomach contents data, using both sets of data collected from turtles in 2016 [29]. For each turtle, we averaged claw and skin ETP values together, and assumed that claw and skin isotopic fractionation are similar. We then assigned each turtle as “empty” if it had no food in its stomach, “herbivorous” if its food bolus was ≥ 75% vegetation (including algae), “omnivorous” if its food bolus was 25–75% vegetation and/or animal matter, and “carnivorous” if its food bolus was ≥ 75% animal matter. We then compared ETP among each category using an ANOVA, independent of species or site, simply to test how long-term ETP, based on isotope data, varied with our assessment of a turtle’s trophic position from stomach contents alone. The analysis is admittedly coarse, but it provides a basic indication of whether turtles are consistently carnivorous, herbivorous, or omnivorous across sites.

Finally, we compared turtle body condition, within sex and species, across the four sites to determine whether overall wetland differences in diet were associated with differences in body condition. We calculated Scaled Mass Indices (SMI) following Peig and Green [28]. We calculated separate SMI for each sex because males and females often exhibit different body condition relationships, but were unable to separate sex in *C. longicollis* because we could not distinguish between sexes [47]. In all calculations, we used straight carapace length as our estimate of “length”. To estimate the mean body lengths of males and females, and the relationships between body mass and length used to calculate SMI [28], we included a broader database of turtle body sizes from the entire southern Murray River basin (27 *C. expansa*, 247 *C.* longicollis, 729 *E. macquarii*; [47]). We then compared SMI across all four wetlands using separate ANOVAs for females and males, within each species (PROC GLM).

We assessed normality and homoscedasticity of variance using Shapiro–Wilk Tests (all *P *> 0.05) and normal probability plots in SAS. All statistical tests were assessed at α = 0.05 and all means are presented ± SE. In mixed models, we determined best-fit covariate structures for random effects using AIC. All main-effects tests were followed by post hoc Tukey tests unless otherwise noted. Raw data can be accessed from Dryad [45].

## Supplementary information

**Additional file 1: Table S1.** Results of full factorial MANCOVA analysis of differences in ETP from claw and skin samples. No effects were significant in the full model.

## Data Availability

The dataset supporting the results of this article is available in the Dryad repository, 10.5061/dryad.z34tmpg8v.

## Abbreviations

AIC: Akaike information criterion
AIR: Air, the international reference standard for nitrogen stable isotopes
ANOVA: Analysis of variance
ANCOVA: Analysis of covariance
ETP: Estimated trophic position
MANOVA: Multivariate analysis of variance
MANCOVA: Multivariate analysis of covariance
PROC GLM: General linear model procedure in SAS
PROC MIXED: Mixed linear model procedure in SAS
SAS: Statistical Analysis System
SCL: Straight carapace length
SE: Standard error
SD: Standard deviation
SMI: Scaled mass index
VPDB: Vienna Pee Dee Belemnite, the international reference standard for carbon stable isotopes

## References

[1] Bearhop S, Adams CE, Waldron S, Fuller RA, Macleod H (2004). Determining trophic niche width: a novel approach using stable isotope analysis. J Anim Ecol.

[2] Bearhop S, Furness RW, Hilton GM, Votier SC, Waldron S (2003). A forensic approach to understanding diet and habitat use from stable isotope analysis of (avian) claw material. Funct Ecol.

[3] Beaupre SJ, Hayes WK, Beaman KR, Cardwell MD, Bush SP (2008). Annual variation in time-energy allocation by timber rattlesnakes (*Crotalus horridus*) in relation to food acquisition. The biology of Rattlesnakes.

[4] Beaupre SJ, Agugliaro J, Van Dyke JU, Zaidan F, Dreslik MJ, Hayes WK, Beaupre SJ, Mackessy SP (2017). Annual energy budgets of the Timber Rattlesnake: advancements, refinements, and open questions. The biology of the Rattlesnakes II.

[5] Cann J (1998). Australian freshwater turtles.

[6] Chessman BC (1983). Observations on the diet of the Broad-shelled Turtle, *Chelodina expansa* Gray (Testudines: Chelidae). Aust Wildl Res.

[7] Chessman BC (1984). Food of the snake-necked turtle, *Chelodina longicollis* (Shaw) (Testudines: Chelidae) in the Murray Valley, Victoria and New South Wales. Aust Wildl Res.

[8] Chessman BC (1986). Diet of the Murray turtle, *Emydura macquarii* (Gray) (Testudines: Chelidae). Aust Wildl Res.

[9] Chessman BC (2011). Declines of freshwater turtles associated with climatic drying in Australia’s Murray–Darling Basin. Wildl Res.

[10] Chessman BC (2019). Effects of temperature and exercise on metabolism of three species of Australian freshwater turtles: implications for responses to climate change. Aust J Zool.

[11] dePersio S, Allender MC, Dreslik MJ, Adamovicz L, Phillips CA, Willeford B (2019). Body condition of eastern box turtles (*Terrapene carolina carolina*) evaluated by computed tomography. J Zoo Wildl Med.

[12] Doi H, Kikuchi E, Takagi S, Shikano S (2007). Changes in carbon and nitrogen stable isotopes of chironomid larvae during growth, starvation and metamorphosis. Rapid Commun Mass Spectrom.

[13] Doi H, Akamatsu F, Gonzalez AL (2017). Starvation effects on nitrogen and carbon stable isotopes of animals: an insight from meta-analysis of fasting experiments. R Soc Open Sci.

[14] Dunham AE, Grant BW, Overall KL (1989). Interfaces between biophysical and physiological ecology and the population ecology of terrestrial vertebrate ectotherms. Physiol Zool.

[15] Estes JA, Riedman ML, Staedler MM, Tinker MT, Lyon BE (2003). Individual variation in prey selection by sea otters: patterns, causes and implications. J Anim Ecol.

[16] Fishelson L, Montgomery LW, Myrberg AH (1987). Biology of surgeonfish *Acanthurus nigrofuscus* with emphasis on changeover in diet and annual gonadal cycles. Mar Ecol Prog Ser.

[17] Galetti M, Rodarte RR, Neves CL, Moreira M, Costa-Pereira R (2016). Trophic niche differentiation in rodents and marsupials revealed by stable isotopes. PLoS ONE.

[18] Gell P, Reid M (2014). Assessing change in floodplain wetland condition in the Murray Darling Basin, Australia. Anthropocene..

[19] Huckins CJF (1997). Functional linkage among morphology, feeding performance, diet and competitive ability in molluscivorous sunfish. Ecology.

[20] Kennett RM, Georges A (1990). Habitat utilization and its relationship to growth and reproduction of the eastern long-necked turtle, *Chelodina longicollis* (Testudinata: Chelidae), from Australia. Herpetologica..

[21] Kingsford RT (2001). Ecological impacts of dams, water diversions and river management on floodplain wetlands in Australia. Aust Ecol.

[22] Layman CA, Arrignton DA, Montana CG, Post DM (2007). Can stable isotope ratios provide for community-wide measures of trophic structure?. Ecology.

[23] Layman CA, Araujo MC, Boucek R, Hammerschlag-Peyer CM, Harrison E, Jud ZR (2011). Applying stable isotopes to examine food-web structure: an overview of analytical tools. Biol Rev.

[24] Leblanc MJ, Tregoning P, Ramillien G, Tweed SO, Fakes A (2009). Basin-scale, integrated observations of the early 21st century multiyear drought in southeast Australia. Water Resour Res.

[25] Liboriussen L, Jeppesen E (2003). Temporal dynamics in epipelic, pelagic and epiphytic algal production in a clear and a turbid shallow lake. Freshw Biol.

[26] Morera-Pujol V, Ramos R, Perez-Mendez N, Cerda-Cuellar M, Gonzalez-Solis J (2018). Multi-isotopic assessments of spatio-temporal diet variability: the case of two sympatric gulls in the western Mediterranean. Mar Ecol Prog Ser.

[27] Olsson OU, Wiktander A, Malmqvist A, Nilsson SG (2001). Variability of patch type preferences in relation to resource availability and breeding success in a bird. Oecologia.

[28] Peig J, Green AJ (2009). New perspectives for estimating body condition from mass/length data: the scaled mass index as an alternative method. Oikos.

[29] Petrov K, Lewis J, Malkiewicz N, Van Dyke JU, Spencer RJ (2018). Food abundance and diet variation in freshwater turtles from the mid-Murray River, Australia. Aust J Zool.

[30] Petrov K. Water regulation and the dispersal of agricultural nutrients: impacts on turtle competition in the Murray River, Australia. Honours thesis, School of Science and Health, Western Sydney University, Richmond, New South Wales, Australia; 2015.

[31] Phillips DL, Inger R, Bearhop S, Jackson AL, Moore JW, Parnell AC (2014). Best practices for use of stable isotope mixing models in food-web studies. Can J Zool.

[32] Post DM (2002). Using stable isotopes to estimate trophic position: models, methods, and assumptions. Ecology.

[33] Pyke GH (1984). Optimal foraging theory: a critical review. Annu Rev Ecol Syst.

[34] Pyke GH, Pulliam HR, Charnov EL (1977). Optimal foraging: a selective review of theory and tests. Q Rev Biol.

[35] Quiggin J (2001). Environmental economics and the Murray–Darling river system. Aust J Agric Resour Econ.

[36] Rosenblatt AE, Heithaus MR (2013). Slow isotope turnover rates and low discrimination values in the American alligator: implications for interpretation of ectotherm stable isotope data. Physiol Biochem Zool.

[37] Scheffer M, Carpenter S, Foley JA, Folke C, Walker B (2001). Catastrophic shifts in ecosystems. Nature.

[38] Seminoff JA, Bjorndal KA, Bolten AB (2007). Stable carbon and nitrogen isotope discrimination and turnover in pond sliders *Trachemys scripta*: insights for trophic study of freshwater turtles. Copeia..

[39] Spencer RJ, Thompson MB, Hume ID (1998). The diet and digestive energetics of an Australian short-necked turtle, *Emydura macquarii*. Comp Biochem Physiol Part A.

[40] Spencer RJ, Georges A, Lim D, Welsh M, Reid AM (2014). The risk of inter-specific competition in Australian short-necked turtles. Ecol Res.

[41] Stephens DW, Krebs JR (1986). Foraging theory.

[42] Terraube J, Arroyo B, Madders M, Mougeot F (2010). Diet specialisation and foraging efficiency under fluctuating vole abundance: a comparison between generalist and specialist avian predators. Oikos.

[43] Vander Zanden MJ, Rasmussen JB (2001). Variation in δ15N and δ13C trophic fractionation: implications for aquatic food web studies. Limnol Oceanogr.

[44] Van Dyke JU, Beaupre SJ (2011). Bioenergetic components of reproductive effort in viviparous snakes: costs of vitellogenesis exceed costs of pregnancy. Comp Biochem Physiol A Mol Integr Physiol.

[45] Van Dyke JU (2020). Dryad.

[46] Van Dyke JU, Ferronato BO, Spencer RJ (2018). Current conservation status of Australian freshwater turtles. Aust J Zool.

[47] Van Dyke JU, Spencer RJ, Thompson MB, Chessman B, Howard K, Georges A (2019). Conservation implications of turtle declines in Australia’s Murray River system. Sci Rep.

[48] Willson JD, Winne CT, Pilgrim MA, Romanek CS, Whitfield Gibbons J (2010). Seasonal variation in terrestrial resource subsidies influences trophic niche width and overlap in two aquatic snake species: a stable isotope approach. Oikos.

[49] Zhou Y, Zhang J, Slade E, Zhang L, Palomares F, Chen J (2008). Dietary shifts in relation to fruit availability among masked palm civets (*Paguma larvata*) in central China. J Mammal.

