# Supplementary material for: Prey-switching does not protect a generalist turtle from bioenergetic consequences when its preferred food is scarce
Source: BMC Ecol. 2020 Feb 18;20:11. doi: 10.1186/s12898-020-00279-6 (PMC7027299; doi:10.1186/s12898-020-00279-6)
Supplement: Supplementary file 1 — Additional file 1: Table S1. Results of full factorial MANCOVA analysis of differences in ETP from claw and skin samples. No effects were significant in the full model. [file 12898_2020_279_MOESM1_ESM.docx]

Prey-switching does not protect a generalist turtle from bioenergetic consequences when its preferred food is scarce

Additional Material

Table S1. Results of full factorial MANCOVA analysis of differences in ETP from claw and skin samples. No effects were significant in the full model.

| Effect | Pillai’s Trace | *F* | Num *df* | Den *df* | *P* |
| --- | --- | --- | --- | --- | --- |
| SCL | 0.002 | 0.11 | 2 | 114 | 0.894 |
| Sex | 0.046 | 2.72 | 2 | 114 | 0.070 |
| SCL*Sex | 0.038 | 2.25 | 2 | 114 | 0.110 |
| Site | 0.023 | 0.45 | 6 | 230 | 0.841 |
| SCL*Site | 0.026 | 0.51 | 6 | 230 | 0.803 |
| Site*Sex | 0.056 | 1.11 | 6 | 230 | 0.360 |
| SCL*Site*Sex | 0.068 | 1.35 | 6 | 230 | 0.236 |
| Species | 0.004 | 0.11 | 4 | 230 | 0.978 |
| SCL*Species | 0.005 | 0.14 | 4 | 230 | 0.967 |
| Species*Sex | 0.052 | 1.53 | 4 | 230 | 0.195 |
| SCL*Species*Sex | 0.064 | 1.91 | 4 | 230 | 0.110 |
| Site*Species | 0.064 | 0.76 | 10 | 230 | 0.665 |
| SCL*Site*Species | 0.067 | 0.80 | 10 | 230 | 0.629 |
